# The Impact of Fetal Growth Restriction on Myocardial Development from Fetal Life to Early Childhood: A Narrative Review

**DOI:** 10.3390/children13030312

**Published:** 2026-02-24

**Authors:** Savina Mannarino, Valeria Calcaterra, Vittoria Garella, Filippo Puricelli, Beatrice Baj, Antonia Quatrale, Cassandra Gazzola, Anna Nosvelli, Irene Raso, Gianvincenzo Zuccotti

**Affiliations:** 1 Pediatric Cardiology Unit, “Vittore Buzzi” Children’s Hospital, 20154 Milan, Italy; savina.mannarino@asst-fbf-sacco.it (S.M.); vittoria.garella@unimi.it (V.G.); filippo.puricelli@asst-fbf-sacco.it (F.P.); beatrice.baj@unimi.it (B.B.); anna.nosvelli@unimi.it (A.N.); irene.raso@asst-fbf-sacco.it (I.R.); 2 Department of Internal Medicine, University of Pavia, 27100 Pavia, Italy; gianvincenzo.zuccotti@unimi.it; 3 Pediatric Department, “Vittore Buzzi” Children’s Hospital, 20154 Milan, Italy; antonia.quatrale@unimi.it (A.Q.); cassandra.gazzola@unimi.it (C.G.); 4 Department of Biomedical and Clinical Science, University of Milano, 20157 Milan, Italy

**Keywords:** fetal growth retardation, intrauterine growth retardation, heart development, myocardium, developmental origins of health and disease

## Abstract

**Highlights:**

**What are the main findings?**

- Fetal growth restriction (FGR) induces early and persistent myocardial remodeling, including reduced cardiomyocyte number, fibrosis, altered ventricular geometry, and subclinical systolic–diastolic dysfunction detectable from fetal life.
- These cardiac and vascular changes can be identified with advanced fetal and neonatal imaging and are associated with increased cardiovascular and metabolic risk from childhood to adulthood.

**What are the implications of the main findings?**

- FGR should be considered an early-life cardiovascular risk condition, requiring a life-course perspective that integrates obstetric, neonatal, and cardiology follow-up.
- Early identification of high-risk fetuses and infants may enable targeted surveillance and preventive strategies to reduce long-term cardiovascular morbidity.

**Abstract:**

**Background/Objectives:** Fetal growth restriction (FGR), historically termed intrauterine growth restriction (IUGR), is a multifactorial condition in which the fetus fails to reach its genetically determined growth potential, most often due to placental insufficiency. Beyond its link with increased perinatal morbidity and mortality, FGR has been associated with long-term cardiovascular risk through early-life programming. The developing fetal heart is vulnerable to chronic hypoxia and nutrient deprivation, potentially inducing structural and functional alterations with lifelong consequences. This narrative review summarizes and critically appraises experimental and clinical evidence on the impact of FGR on myocardial development and cardiovascular health from fetal life to adulthood. **Methods:** We conducted a narrative review using a structured literature search of studies published in the last 15 years in PubMed and Scopus, focusing on experimental, imaging, and epidemiological research evaluating cardiac structure, function, and long-term cardiovascular outcomes in FGR. Evidence from fetal and neonatal echocardiography, including Doppler and speckle-tracking techniques, as well as molecular and histological studies, was examined. No statistical meta-analysis was performed. Results: FGR has been associated with reduced cardiomyocyte number, altered myocardial architecture, increased interstitial fibrosis, and persistent ventricular remodeling. Functional studies suggest early impairments in systolic and diastolic performance, with alterations in cardiac energy metabolism and epigenetic regulation. Advanced imaging may enable detection of subclinical cardiac dysfunction in utero and early postnatally. Epidemiological data suggest an increased risk of hypertension, ischemic heart disease, heart failure, and metabolic disorders in adulthood among individuals born growth-restricted. **Conclusions:** FGR represents an early cardiovascular risk condition. Improved understanding of fetal cardiac programming may help refine risk stratification, surveillance, and preventive strategies to reduce long-term cardiovascular morbidity in individuals born growth-restricted.

## 1. Introduction

Fetal growth restriction (FGR), historically referred to as intrauterine growth restriction (IUGR), is defined as the failure of the fetus to achieve its genetically determined growth potential, most commonly as a consequence of placental insufficiency [1]. Its etiology is multifactorial and includes maternal, placental, and fetal factors, with placental dysfunction representing the dominant mechanism in constitutionally normal fetuses [1,2,3]. Although international societies now prefer the term FGR [4,5,6,7], IUGR remains widely used in clinical practice.

A persistent challenge is the lack of a universal gold-standard definition. FGR must be distinguished from small-for-gestational-age (SGA), a purely statistical category. Many SGA fetuses are constitutionally small, whereas up to 50% of truly growth-restricted fetuses have biometric values above the 10th percentile [8,9,10]. To address this limitation, a Delphi consensus proposed diagnostic criteria combining fetal size with evidence of placental dysfunction, identifying fetuses below the 3rd percentile or with Doppler abnormalities as those at highest risk [7].

The global prevalence of FGR ranges from 3–9% in high-income countries to up to 25% in low- and middle-income settings [3,11]. FGR remains a leading cause of perinatal mortality and morbidity and is associated with both short-term complications and long-term neurodevelopmental, cardiovascular, and metabolic disease, in line with the developmental origins of health and disease hypothesis [12,13,14,15,16,17,18,19]. Early- and late-onset forms reflect different pathogenic mechanisms, with the latter typically characterized by asymmetric growth and brain-sparing hemodynamics [16].

Accurate antenatal diagnosis relies on serial ultrasound assessment of fetal growth, amniotic fluid, and Doppler indices, primarily including umbilical artery and middle cerebral artery measurements to evaluate placental resistance and fetal hemodynamic redistribution; when indicated, additional vessels such as the ductus venosus may also be assessed [20,21,22,23]. When longitudinal data are unavailable, extreme smallness or significant Doppler abnormalities are considered diagnostic, enabling appropriate risk stratification and timing of delivery [24,25].

Among the organs affected by this adverse intrauterine environment, the heart is particularly vulnerable. The human heart undergoes profound structural, functional, and metabolic maturation throughout fetal life and the perinatal period [26,27]. Myocardial development follows a tightly regulated continuum, from early morphogenesis and fetal cardiomyocyte hyperplasia to postnatal hypertrophic growth and metabolic transition toward oxidative phosphorylation [26,27,28,29,30,31]. In parallel, the ventricular myocardium acquires its characteristic helical architecture, which underlies the progressive maturation of myocardial mechanics during gestation and after birth [32,33,34,35,36,37,38,39,40,41,42,43].

This developmental plasticity renders the myocardium highly sensitive to intrauterine stress. In FGR, chronic placental insufficiency, hypoxia, nutrient deprivation, and hemodynamic redistribution occur during a critical window of cardiac maturation, with the potential to permanently alter myocardial structure and function. Human studies have shown early fetal and neonatal alterations in cardiac performance and remodeling in growth-restricted subjects [44], while epidemiological evidence consistently links FGR to increased risk of hypertension, coronary artery disease, heart failure, and metabolic syndrome in adulthood, supporting the concept of fetal cardiac programming [45,46,47].

Despite these observations, current evidence remains fragmented across different ages and methodologies, and key questions persist regarding the mechanisms, temporal evolution, and clinical relevance of cardiac remodeling in FGR.

The aim of this review is therefore to integrate and critically appraise the available data on the impact of FGR on cardiac structure and function from fetal life to adulthood, to identify consistent patterns, highlight knowledge gaps, and provide a pathophysiological framework to support future risk stratification and preventive strategies. By integrating evidence across developmental, experimental, and clinical studies, we aim to provide a unified life-course view of the FGR heart, clarify the links between early myocardial adaptation and later cardiovascular vulnerability, and move toward a more mechanistic and clinically actionable understanding of cardiac programming in fetal growth restriction.

## 2. Methods

This work was conducted as a narrative review based on a structured and transparent literature search strategy. A comprehensive search of the PubMed and Scopus databases was performed to identify relevant studies published in the last 15 years to identify relevant studies addressing the impact of FGR on myocardial development and cardiac structure and function across the life course. Only articles published in English were considered. No formal quality assessment or risk-of-bias analysis was performed, consistent with the narrative design of the review.

The search was performed using the following keywords and their combinations: intrauterine growth restriction, fetal growth restriction, myocardial development, fetal cardiac, placental development, small for gestational age, oxidative stress, and hypoxia. We included original experimental and clinical studies (including observational studies, cohort studies, case–control studies, review, and translational studies) and relevant high-quality review articles when necessary to contextualize specific aspects of myocardial development or pathophysiology. Case reports, small case series, editorials, comments, and opinion papers were excluded. Studies were required to address at least one of the following topics: fetal growth restriction or small for gestational age in relation to myocardial development, cardiac structure or function, placental dysfunction, hypoxia, oxidative stress, or related pathophysiological mechanisms.

**Figure 1:**
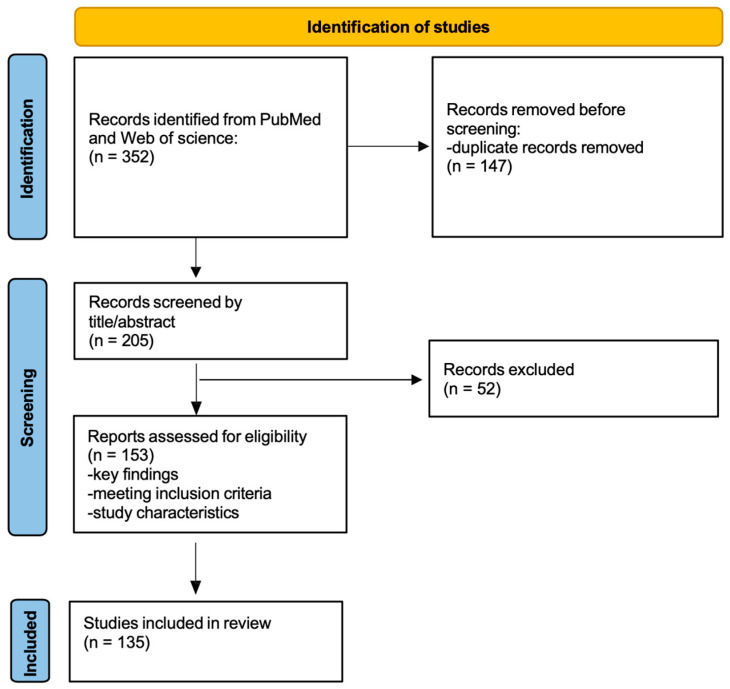
Flowgram of literature selection.

The initial search yielded 352 records. After removal of duplicates and screening of titles and abstracts, 205 articles were considered potentially relevant and selected for further evaluation. One hundred fifty-three articles were retrieved and assessed in full text. Based on predefined inclusion and exclusion criteria, 135 articles were ultimately included in the final qualitative synthesis. The search, screening, and interpretation of the literature were performed by one author and subsequently reviewed by the co-authors, with any uncertainties discussed collectively to reach consensus. Reference lists of included articles were also screened to identify additional relevant publications. All included studies were then used to develop the thematic topics discussed in this review. The literature selection process is summarized in Figure 1. The flow diagram provided is intended to enhance transparency in the study selection process and does not imply a formal PRISMA-guided systematic review methodology.

## 3. Development of the Myocardium Under Physiological Conditions

### 3.1. Fetal Cardiac Ontogeny

Normal cardiac development begins with the formation of the cardiac crescent from mesodermal progenitor cells during early embryogenesis [48]. The definitive four-chambered heart is then established by rightward looping, spatial remodeling, and septation after this crescent fuses at the midline to form a primitive linear heart tube. The atria, ventricles, atrioventricular valves, and outflow tracts are created during this process by coordinated growth and differentiation. Neural crest cells, second heart field progenitors, and epicardial-derived cells are among the cell populations that precisely interact to regulate cardiac morphogenesis, each of which contributes to distinct structural and functional aspects of the heart [48,49]. In order to ensure appropriate temporal and spatial development of the cardiovascular system, these cellular contributions are strictly regulated by intricate genetic networks, signaling pathways (such as Wnt, Notch, BMP, and FGF), and environmental factors. Disruption of any of these processes can result in congenital heart defects, highlighting the intricate and highly coordinated nature of cardiac development [48,49].

Cardiomyocyte proliferation is strong in the early stages of pregnancy, when cells are primarily mononucleated and heavily dependent on glycolytic metabolism. This proliferative phase is critical for establishing adequate cardiomyocyte endowment. As gestation advances, cardiomyocytes transition toward hypertrophic growth and binucleation, representing a shift from hyperplastic to hypertrophic growth patterns [15,50]. The fetal heart is remarkably flexible, able to change both structurally and functionally in response to physiological and environmental stressors. In chronic hypoxemia or nutrient deprivation, such as in placental insufficiency or maternal malnutrition, the myocardium undergoes geometric remodeling to preserve cardiac output and maintain perfusion to vital organs. This involves changes in chamber dimensions, wall thickness, and myocardial fiber orientation, optimizing pumping efficiency under suboptimal conditions. Increased wall thickness can enhance contractile force despite limited oxygen, while ventricular geometry adjustments help balance the hemodynamic load in parallel fetal circulation. These structural adaptations support the fetal heart’s resilience even under prolonged in utero stress. They are accompanied by cellular and molecular changes, such as modified cardiomyocyte proliferation, extracellular matrix remodeling, and changes in metabolic pathways [51].

### 3.2. Maturation of the Myocardium from Fetus to Neonate

The transition from fetal to neonatal life represents one of the most profound physiological challenges encountered by the cardiovascular system. In utero, circulation is characterized by right ventricular dominance and parallel blood flow supported by the ductus arteriosus and foramen ovale. At birth, closure of these shunts and the abrupt increase in systemic vascular resistance necessitate rapid functional reorganization, with the left ventricle assuming systemic dominance [52,53]. This hemodynamic transition is accompanied by substantial metabolic remodeling. Cardiomyocytes shift from a predominantly glycolytic metabolism toward oxidative phosphorylation, supported by increased mitochondrial content and maturation of oxidative enzymes. These changes are essential to meet the heightened energetic demands of postnatal life [53,54]. Structurally, postnatal maturation involves the organization of myofibrils, expansion of the sarcoplasmic reticulum, and development of mature intercalated discs, all of which enhance electrical coupling and contractile efficiency. Nevertheless, the neonatal myocardium remains functionally distinct from that of the adult heart. Reduced calcium-handling capacity, shorter sarcomeres, and limited contractile reserve render the neonatal heart more sensitive to alterations in preload, afterload, and oxygen availability. These features reflect both developmental immaturity and adaptive flexibility during a critical period of physiological transition [52].

### 3.3. Structural and Functional Differences Compared to Adults

Neonatal myocardium differs fundamentally from adult myocardium in structural, cellular, and functional characteristics. Neonatal hearts have a higher proportion of mononucleated cardiomyocytes, which retain proliferative capacity, whereas adult cardiomyocytes are largely terminally differentiated. Neonates have lower mitochondrial density, which supports a metabolic profile that depends more on lactate and glycolysis than on the oxidative phosphorylation that is common in adults. Functionally, neonatal myocytes exhibit shorter sarcomeres, reduced calcium-handling efficiency, and lower contractile force. These adaptations allow the neonatal heart to meet the unique circulatory demands after birth while remaining flexible to changes in preload, afterload, and oxygen availability [50,54]. The neonatal heart exhibits also reduced compliance and distinct force-frequency relationships compared to the adult heart, reflecting the immaturity of its contractile machinery. These functional differences are largely due to underdeveloped calcium handling mechanisms, including limited sarcoplasmic reticulum capacity and immature expression of calcium transport proteins, which constrain the heart’s ability to modulate contraction strength and relaxation efficiently. As a result, neonatal myocardium relies more heavily on extracellular calcium influx for excitation–contraction coupling, and its response to changes in heart rate differs significantly from that of adults. These characteristics underscore the unique physiological adaptations of the heart during the early postnatal period, highlighting both its vulnerability and its capacity for developmental plasticity [52].

The contractile apparatus of the neonatal heart differs markedly from that of the adult heart, with neonatal cardiomyocytes expressing distinct isoforms of myosin heavy chains and titin. These isoforms contribute to differences in contractile velocity, force generation, and elasticity, reflecting the developmental stage of the myocardium. Neonatal myocytes also exhibit less organized sarcomere structure and a higher proportion of compliant titin isoforms, which together influence myocardial stiffness and the ability to handle varying preload and afterload conditions. These molecular and structural distinctions are critical for the adaptive function of the early postnatal heart and may have implications for susceptibility to stress or pathological conditions later in life [53]. These molecular differences contribute to distinct mechanical properties, including reduced contractile force generation and altered length-tension relationships. Additionally, the neonatal heart demonstrates greater regenerative capacity, with cardiomyocytes retaining proliferative potential that is largely lost in adult hearts [50].

## 4. Pathophysiology of IUGR

Normal fetal growth depends primarily on an adequate supply of oxygen and nutrients through the placenta. There are several causes of IUGR, including maternal, fetal, and placental factors [55]. Genetic factors also play an important role, and several maternal, fetal, and placental genes have been identified in the etiology of IUGR [55]. In high-income countries, fetal growth restriction is mainly caused by placental insufficiency, unlike in low- and middle-income countries, where maternal malnutrition is the main cause of IUGR [55]. The placenta is essential for the efficient transport of nutrients and oxygen from the mother to the fetus and consequently for maintaining normal fetal growth. The placenta of a pregnancy characterized by IUGR is smaller than normal placentas throughout gestation and exhibits abnormal development of both the placental villi and the fetal vasculature within them [23]. Trophoblasts, which are specialized epithelial cells, show abnormal function and development in the placentas of IUGR pregnancies. This then leads to an altered balance between proliferation and apoptotic death, premature cellular senescence, and reduced colonization of maternal decidual tissue. Thus, the placenta undergoes aberrant alterations at the macroscopic and cellular levels in fetal growth restriction, which can limit fetal exchange capacity and downstream growth [56]. In humans, efficient placental exchange is made possible by the branched villous tree structure of the placenta. Each villus is composed of a mesenchymal nucleus enclosed by a trophoblastic bilayer, which serves as the main barrier to exchange, and an inner layer of mononuclear cytotrophoblasts that fuse to form the overlying syncytiotrophoblast or differentiate into a third lineage called the extravillous trophoblast. The extravillous trofloblast invades the decidua to reshape the maternal spiral arteries into non-vasoactive conduits and ensure constant perfusion of the placenta by maternal blood throughout pregnancy [57]. Placentas in IUGR pregnancies exhibit altered development of both the placental villi and the fetal vasculature within them [58]. As gestation progresses, the volume and surface area of terminal and intermediate villi in normal pregnancies increase through continued villous germination to maximize exchange efficiency and maintain fetal growth. This does not occur in IUGR pregnancies, in which placentas show a reduced number of all villous subtypes, with thinner and longer terminal villi, characterized by less branching and budding [59]. Capillary loops in the terminal villi of IUGR placentas also exhibit reduced density, elongation, branching, and less spiralization than placentas of normally grown fetuses [60]. This is of particular importance since the non-muscled intermediate and terminal peripheral villi constitute the majority of the total placental volume in the third trimester and serve as primary sites of exchange. Other vascular changes in FGR placentas include reduced or absent vascular lumens in intermediate and terminal villi, loss of CD34 endothelial marker expression, and filling of pre-existing vascular spaces with CD34-negative cells [61]. These alterations in villous structure and vascularization suggest that the pathophysiology of IUGR begins early in placental development and compromises both the villus and capillary area, limiting exchange capacity. The oxygen diffusion potential is compromised in about half in IUGR placentas, resulting in approximately one-third lower than in normal placentas [62]. Normal fetal development depends on the availability of respiratory gases, water, ions, and nutrients, which in turn depend on villous expansion and the nutrient-carrying capacity of the syncytiotrophoblast. Amino acid transport is crucial for protein synthesis, and reduced amino acid uptake can limit fetal growth. In placentas from IUGR pregnancies, a reduction in the expression and activity of trans-membrane transporters has been documented in both the apical and basal plasma membranes of the syncytiotrophoblast [63,64].

Maternal factors that can lead to IUGR are mainly chronic maternal diseases, such as arterial hypertension, chronic kidney disease, autoimmune diseases, genetic predispositions, particularly mutations affecting genes such as MTHFR, ESR1, TNFalpha, FOXD1, ET-1, all of which affect vascular proliferation, coagulation, and chronic inflammation [63]. Drug use during pregnancy or chronic malnutrition in women can also lead to IUGR. An example is endothelin 1 (ET-1), the most potent vasoconstrictor factor. The pathogenesis of placental vasoconstriction is unknown, but a hyperactivating mutation of ET-1 can lead to altered placental development and thus IUGR. Arslan et al. [65] enrolled 25 pregnant IUGR women and 19 controls in their study; mean maternal ET-1 and mean fetal ET-1 concentrations were significantly higher in IUGR patients. Quintero-Renderos et al. [63]. demonstrated the central role of Forkhead Box D1 (FOXD1) mutations in recurrent miscarriage and IUGR pathogenesis via regulation of complement C3 and placental growth factor (PlGF) and described a functional link between FOXD1 and implant and placental diseases. Golovchenko et al. [66] also identified the TG haplotype (rs2234693-rs9340799 polymorphisms) of the estrogen receptor α (ESR1) gene as a risk factor for the development of IUGR. ESR1 is a ligand-activated transcription factor, essential for appropriate hormonal binding. During pregnancy, estrogen stimulates the activation of the IGF-1 receptor, promoting fetal growth and the response to various signals, such as hypoxia, hormones, and nutrients. TNF-α also participates in the inflammatory response, the induction of apoptosis, cell proliferation, differentiation and migration [67]. Finally, maternal pathological conditions such as pre-eclampsia are also related to IUGR, likely because the pathogenic mechanism of the two pathological conditions is very similar and based on inadequate trophoblast invasion into the maternal spiral arteries and maternal endothelial dysfunction [68].

Fetal causes of growth retardation include chromosomal defects, monogenic syndromes, and abnormal methylations [69]. Chromosomal abnormalities, most frequently aneuploidy, have been reported in approximately 19% of fetuses with IUGR. Significant fetal growth restriction is often observed with trisomy 13, trisomy 17 [70], and trisomy 18, which are usually easily detected on ultrasound for the presence of multiple malformations [71]. The other genetic disorders that lead to IUGR are Cri-du-chat syndrome [72], resulting from a deletion of the short arm of chromosome 5, and Williams-Beuren syndrome, caused by a heterozygous deletion in the region of chromosome 7q11.23 [73]. IUGR can also result from a monogenic disorder, typically abnormalities of genes associated with fetal growth, short stature, or other skeletal abnormalities (such as skeletal dysplasia) [74]. Most of these syndromes cause short stature, but many of them are also associated with intellectual disability. Some genetic syndromes cause symmetric IUGRs, in which head circumference and long bones are equally affected: examples are Cornelia de Lange syndrome (the most common mutation in the NIPBL gene), Smith–Lemli–Opitz (mutation in the DHCR7 gene), and Meier–Gorlin (mutation in the ORC1, ORC4, ORC6, CDT1, or CDC6 genes). Other genetic syndromes cause asymmetric IUGRs, such as Noonan syndrome (most common mutation in the PTP11 gene), achondroplasia, or hypochondroplasia (mutation in the FGFR3 gene) [75].

All these placental, maternal, and fetal mechanisms ultimately converge in creating a chronic intrauterine environment characterized by hypoxia and reduced nutrient availability. This hostile milieu not only limits overall fetal growth, but also represents the pathophysiological basis for the profound cardiovascular adaptation observed in IUGR fetuses. In particular, the fetal heart is forced to operate under conditions of chronic oxygen and substrate deprivation, leading to metabolic reprogramming, structural and functional remodeling, and altered cardiomyocyte maturation. These early alterations in cardiac development and metabolism constitute a key component of fetal programming and provide the mechanistic link between the etiological factors of IUGR and the increased susceptibility to cardiovascular disease later in life, as discussed in the following section [76,77].

## 5. Effects of Intrauterine Growth Restriction on the Myocardium

### 5.1. Structural Alterations

FGR induces profound structural remodeling of the developing heart, characterized by increased relative wall thickness, altered ventricular geometry, reduced chamber compliance, and a permanent reduction in total cardiomyocyte number that persists into adulthood [51,78,79]. At the cellular level, delayed cardiomyocyte differentiation is associated with transient compensatory proliferation of mononucleated cells but also with increased fetal apoptosis, resulting in an overall deficit in cardiomyocyte endowment. This reduced cell number contributes to exaggerated compensatory hypertrophy and disorganized myocardial architecture later in life [50,80].

Fibrosis represents a prominent feature of FGR-affected hearts, characterized by increased interstitial collagen deposition and upregulation of key profibrotic genes, including TGF-β and CTGF. This fibrotic remodeling contributes to myocardial stiffness, impaired compliance, and altered ventricular filling, which can compromise overall cardiac function [81]. Notably, these changes are more pronounced in the left ventricle, which also exhibits reduced capillary density, despite evidence of increased vascular cross-sections in some experimental models, suggesting a mismatch between myocardial growth and vascular development. The differential remodeling between the left and right ventricles has been well documented, with left ventricular alterations, such as heightened fibrosis, changes in extracellular matrix composition, and structural disorganization, being particularly prominent. These adaptations likely reflect the left ventricle’s greater hemodynamic workload and its critical role in maintaining systemic perfusion, highlighting how IUGR can induce region specific myocardial vulnerability with potential long-term functional consequences [80]. These chamber-specific differences are biologically plausible given the distinct loading conditions of the fetal circulation: in utero, the right ventricle is functionally dominant and exposed to higher afterload, whereas after birth the left ventricle becomes the systemic pump and undergoes a rapid increase in workload. Available studies in FGR generally describe biventricular remodeling, although left ventricular structural changes appear more pronounced [51,78,79,80]; direct human evidence comparing cardiomyocyte-level differences between the two ventricles remains limited and warrants further investigation.

### 5.2. Functional Changes

FGR hearts demonstrate both systolic and diastolic dysfunction that begins in utero and often persists into the postnatal period, reflecting the lasting impact of intrauterine stress on cardiac structure and function [51,79]. Systolic dysfunction is evidenced by reduced ejection fraction, decreased myocardial performance index, and persistently diminished left ventricular torsion and strain, highlighting impaired contractile capacity even in otherwise normotensive models [78,81].

Despite these impairments, cardiac output may be paradoxically elevated as a compensatory response, particularly in the fetal period. Diastolic dysfunction is particularly prominent, with impaired myocardial relaxation and reduced compliance manifesting as decreased mitral and tricuspid E′ velocities on tissue Doppler imaging and elevated E/E′ ratios. These changes reflect altered calcium handling, increased myocardial stiffness from fibrosis, and intrinsic cardiomyocyte dysfunction. The diastolic impairment appears to be an early and sensitive marker of FGR-related cardiac dysfunction [78,79].

Moreover, energy metabolism is profoundly disrupted in FGR hearts. There is decreased expression of key substrate transporters, including glucose transporter GLUT4 and fatty acid transporters CD36 and FATP. This is accompanied by reduced mitochondrial abundance and lower levels of electron transport chain complexes (particularly complexes II and IV), leading to impaired ATP production. The altered metabolic profile suggests that IUGR hearts have diminished capacity for the critical metabolic switch from carbohydrate to fatty acid oxidation that normally occurs around birth [54,80,82].

### 5.3. Histopathological and Molecular Evidence

Ultrastructural analyses reveal key alterations in the contractile machinery of FGR hearts. Cardiomyocytes show shorter sarcomeres and M-band abnormalities associated with impaired function, along with disrupted spatial organization of energetic units, characterized by increased distance between mitochondria and myofilaments that may hinder efficient energy transfer. Gene expression profiling indicates coordinated upregulation of pathways related to oxygen homeostasis, oxidative phosphorylation, and NADH dehydrogenase activity, reflecting persistent energetic and structural remodeling [80]. Altered expression of myosin heavy chain isoforms, titin variants, and stress markers such as atrial natriuretic peptide further supports this reprogramming [81].

These changes suggest fundamental reprogramming of cardiac energetics that may compromise the heart’s ability to meet metabolic demands, particularly during periods of stress [54,80]. Epigenetic modifications likely play a central role in mediating the long-term cardiovascular consequences of FGR. The adverse intrauterine environment induces gene modifications through mechanisms including DNA methylation, histone modifications, and altered microRNA expression, resulting in cardiovascular programming that persists into adult life [83,84]. This epigenetic programming may explain why the structural and functional cardiac abnormalities observed in FGR fetuses persist postnatally and contribute to increased cardiovascular disease risk in adulthood [15,85].

These structural, functional, and metabolic changes represent the core myocardial remodeling phenotype associated with FGR and provide the mechanistic basis for the clinical findings described in the following sections.

## 6. Cardiac and Hemodynamic Assessments in FGR

The hemodynamic fetal responses secondary to placental insufficiency are characterized by selective changes in peripheral vascular resistance that allow preferentially delivering of nutrients and oxygen to vital organs (i.e., brain, myocardium and adrenal glands) while reducing the perfusion to less vital organs (primarily kidneys, gastrointestinal tract and lower extremities), with the purpose of compensating for the diminished resources [86,87]. At the same time, the fetal heart adapts with changes in size, shape and function. In the early stages, the brain supply of substrates and oxygen can be maintained at near-normal levels despite any absolute reduction in placental transfer. With the progressive deterioration of fetal conditions, protective mechanisms are overwhelmed by the fall of cardiac output and fetal distress occurs [86,88].

Evaluation of placental and fetal blood flows, and fetal cardiac function is crucial to interpret fetal adaptation to hypoxia, to predict prognosis, and to ensure appropriate clinical management of compromised fetuses [2,89,90].

### 6.1. Early-Onset FGR

Doppler velocimetry of the umbilical artery (UA) provides a good assessment of intrauterine fetal well-being. In early-onset FGR, abnormal UA Doppler parameters are a hallmark sign of placental insufficiency, with an increased UA pulsatility index often preceding absent or reversed end-diastolic flow as placental resistances increase [91,92,93].

In growth-restricted fetuses, Doppler velocimetry of the middle cerebral artery (MCA) and the cerebroplacental ratio (CPR), calculated as the ratio between MCA and UA pulsatility indexes, identify the so-called “brain sparing effect” which is associated with adverse perinatal outcomes [94,95,96]. It should be emphasized that vascular adaptation is limited and soon a plateau corresponding to the nadir of the pulsatility index is reached; therefore, arterial vessels appear unsuitable for longitudinal monitoring of growth-restricted fetuses [97].

As mentioned, hemodynamic changes aim at a preferential shift in cardiac output in favor of the left ventricle leading to improved perfusion to the brain. To achieve this goal, the left ventricle (LV) afterload is reduced by cerebral vasodilation, whereas the right ventricle (RV) afterload results increased due to pulmonary, splanchnic and placental vasoconstriction. This results in increased aortic and decreased pulmonary time-to-peak velocity and in a relative increase in left cardiac output (LCO) and decrease in right cardiac output (RCO) [86,98].

With deterioration of fetal conditions, ventricular ejection fraction and cardiac output may be significantly and symmetrically decreased at the level of both ventricles, reflecting progressive impairment of myocardial contractile function resulting from chronic hypoxemia and exhaustion of early compensatory mechanisms [99]. These fetuses also show impaired ventricular filling with lower trans-tricuspid and trans-mitral E/A peak velocities ratio compared to normally grown fetuses, with early impairment of RV filling [100,101]. Studies in the fetal venous circulation have demonstrated that fetal deterioration leads to increase in right atrial pressure with increase in venous reverse flow during atrial contraction at the level of the inferior vena cava (IVC), that progressively and subsequently extent to the ductus venous (DV) and finally to the umbilical vein (UV). Development of reverse flow in the DV and UV is typically associated with rapid onset of fetal heart rate anomalies, particularly when associated with visualisation of higher velocities in coronary blood flow [102,103]. At this stage, reduced or reverse end-diastolic velocities may also be present in pulmonary veins [102]. There is also some evidence showing a relationship between aortic isthmus (AoI) Doppler features, that may indicate changes in peripheral and cerebral resistances, and UA and DV Doppler changes; however, controversial results have emerged from the studies evaluating such correlation [104].

At the moment, the only available randomized controlled trial evaluating early-onset FGR monitoring is the Trial Randomizing Umbilical and Fetal Flow in Europe (TRUFFLE) [105]. This study has demonstrated that expectant management and delayed delivery are acceptable in FGR fetuses as long as a reduced short-term variation at continuous cardiotocography (cCTG) or early-stage DV Doppler changes are present alone, but not if both are present in association or in case of documented late-stage DV Doppler abnormalities [106,107].

### 6.2. Late-Onset FGR

In late-onset FGR, hemodynamic changes result usually more subtle and cardiovascular adaptation appears somehow different from early-onset disease. Although the burden of perinatal complications is lower compared to early-onset disease, late FGR is associated with an increased risk of adverse short- and long-term outcomes, including hypoxemic events and neuro-developmental delay compared to normally grown fetuses [108].

UA Doppler velocimetry results frequently normal, whereas abnormal MCA Doppler velocimetry or altered CPR are associated with admission to neonatal intensive care at birth and increased risk of perinatal complications [109].

The myocardium has been shown to develop a variety of structural and functional changes defined as cardiac remodelling. Cardiac afterload appears minimally affected, so that left and right cardiac output and their ratio appear not significantly different from normally grown fetuses when corrected for fetal weight [110]. To maintain an adequate stroke volume with less contraction force and to reduce wall stress to better tolerate pressure overload, these hearts develops a more spherical shape that is usually quantified by the ventricular sphericity index (SI), calculated as the ratio of ventricular length (base-to-apex diameter) to ventricular width (maximum transverse diameter) measured at end-diastole. Fetuses with cardiac remodelling did not show significant differences in baseline clinical and Doppler characteristics but were shown to develop a poorer short-term perinatal outcome [111].

A randomized study is needed to address optimal monitoring strategy and timing of delivery of late FGR fetuses. The ongoing TRUFFLE study and TRUFFLE 2 study are likely to provide further insights into the actual role of fetal cardiac and hemodynamic parameters [112].

Additional parameters are currently being studied and are expected to help defining the optimal monitoring strategy and time of delivery of FGR fetuses. Among these, the myocardial performance index (MPI) and the fetal heart quantitative technique (fetal HQ) appear promising approaches.

MPI is defined as the sum of the isovolumic contraction and relaxation times divided by the ejection time, and it has been shown to represent a reliable parameter for global ventricular function. An abnormal increased MPI has been shown in FGR compared to appropriately grown fetuses in the early stages of cardiac adaptation, prior to detection of UA Doppler changes [113,114].

Fetal HQ is a new technology specifically designed for fetal cardiac quantitative assessment, utilizing speckle tracking imaging. This technique has shown overall smaller dimensions and a more spherical shape of FGR hearts compared to normal fetuses and that in the early stages these hearts exhibit increased RV fractional area change, significantly higher LV fractional shortening and altered strain parameters, likely as result of the compensatory mechanisms that counteract nutrient and oxygen deprivation [115,116,117].

## 7. Clinical Cardiovascular Consequences

Building on the structural and functional mechanisms described in Section 5, the following section focuses on the clinical manifestations of FGR-related cardiovascular remodeling across the life course.

### 7.1. Early Neonatal Complications

The transition from fetal to neonatal circulation requires rapid and complex cardiovascular adjustments, including marked changes in preload, afterload, and systemic vascular resistance, placing a substantial functional demand on the neonatal heart. In this context, the heart plays a central role in postnatal adaptation, and even subtle impairments in cardiac function or structure may have relevant clinical implications [45].

Growing evidence indicates that infants with FGR frequently exhibit early cardiovascular involvement, which may remain clinically silent but can be detected using sensitive echocardiographic and biochemical markers. Studies have reported signs of cardiac stress and impaired myocardial performance already in the first days of life [118,119], as well as early structural cardiac remodeling detectable at birth [120], suggesting that cardiovascular dysfunction is not an isolated finding but a common component of the neonatal phenotype associated with intrauterine growth restriction. Within a broader clinical perspective, these observations are consistent with the concept that early cardiovascular alterations in growth-restricted infants may reflect prenatal programming with potential implications beyond the neonatal period [14,121]. Understanding the nature and extent of these early cardiovascular alterations is therefore crucial to interpret immediate postnatal complications and to identify infants who may benefit from closer monitoring and follow-up.

In the first postnatal days, neonates with intrauterine growth restriction may exhibit early signs of cardiac stress, as evidenced by elevated BNP concentrations, together with subtle alterations in global cardiac function, including subclinical biventricular dysfunction detectable through an increased myocardial performance index (MPI), also known as the Tei index, as reported by Fouzas et al. [119]. The myocardial performance index is a Doppler-derived parameter that integrates systolic and diastolic time intervals, providing a measure of global ventricular function. In this context, Zaharie et al. demonstrated that left ventricular systolic dysfunction, defined by an MPI greater than 0.47, was present in approximately 40% of IUGR newborns, compared with 4.7% of appropriate-for-gestational-age controls, indicating a high prevalence of early functional impairment [118].

Beyond conventional echocardiographic measures such as MPI and ejection fraction, advanced parameters of myocardial function have increasingly been employed to detect subtle cardiac alterations in this population. Using speckle-tracking echocardiography, these approaches allow the identification of subclinical myocardial dysfunction not only at birth but also during the first months of postnatal life. Accordingly, reported that while left ventricular longitudinal strain (LVLS) values were comparable between FGR infants and controls at birth, control infants exhibited a significant postnatal increase reflecting normal functional maturation, whereas IUGR infants failed to show a similar improvement, resulting in significantly lower LVLS at 3–4 months of age [122].

With regard to cardiac morphology, complementary evidence indicates that newborns with intrauterine growth restriction exhibit significantly smaller left heart dimensions at birth, consistent with early cardiac remodeling. Zaharie et al. described reduced left ventricular and left atrial dimensions in FGR infants compared with controls, with a postnatal increase in left ventricular size that partially, but not completely, reduced the morphological gap during follow-up [118]. Similar alterations in left ventricular geometry were reported by Bjarkø et al., supporting the concept of fetal cardiovascular adaptation to an adverse intrauterine environment [120].

Quantitative evidence of early vascular remodeling in neonates with intrauterine growth restriction has been provided by Skilton et al. [55], who demonstrated a significantly increased aortic intima–media thickness in growth-restricted newborns compared with appropriate-for-gestational-age controls.

This finding indicates that structural arterial changes are already present in the immediate postnatal period and supports the concept that adverse intrauterine conditions directly affect vascular development. In line with the fetal programming hypothesis originally proposed by Barker [121], early arterial wall thickening may represent a substrate for long-term cardiovascular vulnerability. Although direct neonatal measurements of arterial stiffness are limited, human studies in individuals born with IUGR have consistently reported reduced arterial distensibility and compliance, indicative of a stiffer arterial phenotype [123].

These findings indicate that vascular and cardiac alterations coexist at birth, creating an early postnatal hemodynamic profile characterized by increased arterial stiffness and cardiac workload, which may contribute to the heightened vulnerability observed in growth-restricted neonates.

### 7.2. From Fetal Cardiovascular Remodeling to Outcomes in Childhood, Adolescence, and Adulthood

FGR involves fetal nutritional restriction during key windows of epigenetic programming, particularly from mid-gestation through the late third trimester, leading to adaptive “thrifty” developmental pathways suited to low nutrient availability. When postnatal nutrition is normal or excessive, this mismatch can become maladaptive, predisposing individuals to metabolic disorders, such as obesity, type 2 diabetes, and metabolic syndrome, that further increase long-term cardiovascular risk.

This pathophysiological process is further accentuated postnatally by compensatory growth, termed “catch-up growth,” representing accelerated somatic growth aimed at restoring intrauterine growth deficits. Paradoxically, although catch-up growth normalizes body weight and anthropometrics, it may exacerbate adverse cardiometabolic outcomes, potentiating cardiovascular risk.

Within the DOHaD framework, the myocardial and vascular alterations can be interpreted as early manifestations of prenatal cardiovascular programming [121,124]. The remodeling phenotype described above appears to persist from infancy through adulthood, with structural and functional abnormalities documented across the life course.

The study “Cardiovascular Risk in Young Finns”, analyzed a longitudinal cohort of 3596 participants followed from childhood into adulthood, and demonstrated that individuals born SGA exhibited significantly elevated triglycerides, LDL cholesterol, and systolic blood pressure at age 31, along with increased carotid intima-media thickness (IMT), relative to participants born AGA [125]. These findings indicate that IUGR induces early cardiovascular imprinting, associated with subclinical endothelial dysfunction and atherogenic phenotypes detectable in young adulthood, potentially predictive of clinical CVD in later life.

Accordingly, early myocardial and vascular alterations observed from infancy may reflect a programmed cardiovascular phenotype predisposing to later hypertension, cardiomyopathy, and heart failure [121,124].

In the “Enigma” study [126] 882 young adults (mean age 21 years) were enrolled, and birth weight was evaluated in relation to cardiovascular parameters including brachial and central blood pressure, augmentation index (AIx), aortic pulse wave velocity (PWV), cardiac output, and stroke volume, alongside anthropometric measurements. Initial analyses demonstrated that low birth weight was associated with higher brachial systolic blood pressure, central pulse pressure, and AIx, while in females there was a positive correlation between birth weight and cardiac output and stroke volume. However, in a subsequent analysis adjusted for body surface area (BSA), these associations were attenuated or rendered non-significant, suggesting that observed hemodynamic differences in adulthood may primarily reflect body size rather than intrinsic alterations due to low birth weight.

A binational cohort study encompassing over 3.4 million births in Sweden and Denmark examined the relationship between SGA, prematurity, and cardiovascular risk in early adulthood [127]. During a median 10-year follow-up from age 18, 29,742 individuals experienced cardiovascular events, including hypertension, coronary artery disease, and cerebrovascular events. Compared to AGA term-born individuals (10th–90th percentile; 39–40 weeks gestation), SGA participants demonstrated a 38% increased risk of cardiovascular disease (hazard ratio, HR = 1.38; 95% CI: 1.32–1.45), whereas preterm-born individuals had a 31% increased risk (HR = 1.31; 95% CI: 1.25–1.38). Sibling analyses controlling for shared familial factors reduced the HR for SGA to 1.11 (95% CI: 0.99–1.25), indicating that a portion of the SGA-associated risk may be attributable to genetic and familial environmental factors. In contrast, the impact of prematurity remained significant in sibling analyses (HR = 1.21; 95% CI: 1.07–1.37), suggesting an independent effect on cardiovascular risk.

These findings support the concept that prenatal programming interacts with postnatal modifiers, and that the clinical expression of cardiovascular risk may depend on additional “second hits”, including lifestyle factors, metabolic stress, and environmental exposures encountered later in life [128].

These results confirm that SGA status and prematurity are independently associated with increased cardiovascular risk in early adulthood. In addition to IUGR, prematurity independently contributes to cardiac remodeling, elevated blood pressure, and insulin resistance [120,129]. The most pronounced effects are observed in preterm individuals with concomitant IUGR, whereas preterm infants without IUGR are comparable to controls. Even in the first days of life, preterm-IUGR neonates exhibit higher systolic blood pressure and increased aortic intima-media thickness relative to preterm-AGA infants [129].

Studies specifically evaluating IUGR and cardiovascular risk are limited to smaller populations due to the reasons described above.

In a follow-up study of 19 young adults born at term with IUGR and 18 controls, assessed by echocardiography, carotid echo-tracking, tonometry, blood pressure measurement, and laser Doppler, ascending aortic diameter was significantly reduced in IUGR subjects, even after adjustment for BSA, whereas left ventricular diameter was smaller only prior to adjustment. AIx was significantly elevated in the IUGR cohort, while common carotid diameter, intima-media thickness ratio, carotid distensibility, and left ventricular mass and function did not differ between groups. IUGR status remained an independent predictor of ascending aortic diameter. These findings indicate that IUGR secondary to placental dysfunction may lead to reduced aortic dimensions and enhanced systolic pressure amplification in adulthood, with potential adverse consequences for future left ventricular performance due to increased aortic impedance [129].

In a study [130] involving 7-year-old children born very preterm with IUGR, non-invasive assessments were performed to evaluate blood pressure, cardiac dimensions and function, the diameters, distensibility, and stiffness of the abdominal aorta, carotid, and popliteal arteries, endothelial function, and carotid intima-media thickness. Controls included preterm-AGA children, matched for age and sex. Preterm-IUGR children demonstrated reduced microvascular response to acetylcholine, a marker of early endothelial dysfunction, lower aortic stiffness, and higher distensibility compared with preterm-AGA children, potentially reflecting an initial adaptive mechanism, alongside reduced carotid IMT relative to term-AGA children. The preterm-AGA cohort exhibited the highest aortic β values and lowest distensibility, while height-corrected systolic blood pressure was elevated in both preterm groups compared with term-AGA children. No significant differences in cardiac size or function were observed across groups. These findings suggest that IUGR in the context of very preterm birth is associated with early structural remodeling of the arterial wall, whereas prematurity alone is linked to elevated systolic blood pressure without detectable changes in cardiac morphology or function by conventional echocardiography.

Tzschoppe et al. [131] compared IUGR children with AGA children, evaluating anthropometric, cardiovascular, and metabolic parameters, including LDL cholesterol, insulin, leptin, IGF-I, DHEAS, carotid IMT, blood pressure, and subcutaneous fat mass, up to 6 years of age. All IUGR children experienced catch-up growth, but at 6 years BMI SDS and subcutaneous fat mass remained slightly lower than in AGA controls. Leptin concentrations were significantly lower in IUGR children, an early indicator of metabolic vulnerability, while IGF-I, insulin, LDL, and DHEAS were comparable. Despite this, mean cIMT was significantly higher in IUGR children, representing a subclinical marker of vascular remodeling and early atherosclerosis, whereas mean blood pressure did not differ between groups.

The main human studies investigating cardiovascular outcomes in individuals born with FGR/SGA are summarized in Table 1.

## 8. Future Perspectives

### 8.1. Preventive and Therapeutic Strategies

#### 8.1.1. Antenatal Prevention

Preventive strategies during pregnancy represent an important opportunity to mitigate the adverse fetal cardiovascular programming associated with FGR. At present, low-dose aspirin remains the only intervention with consistent clinical evidence supporting its use for the prevention of placental-mediated complications. When administered at doses ≥100 mg and initiated at or before 16 weeks of gestation in high-risk pregnancies, aspirin reduces the incidence of small-for-gestational-age births by approximately 10%, likely through partial improvement of spiral artery remodeling and attenuation of placental ischemia–reperfusion injury [134].

Maternal lifestyle factors may also modulate placental and fetal cardiovascular function. Higher levels of maternal physical activity during the first and second trimesters are associated with a lower risk of FGR, and moderate prenatal exercise does not appear to adversely affect fetal heart rate or uteroplacental blood flow, while being associated with modest improvements in umbilical artery Doppler indices [134].

Considerable interest has recently focused on antioxidant strategies. In experimental models, maternal administration of antioxidants such as N-acetylcysteine and melatonin has been associated with reductions in placental vascular resistance, improvement of endothelial function, mitigation of coronary stiffness, and increased myocardial tolerance to ischemia–reperfusion injury in the offspring [134,135]. Although a substantial proportion of preclinical studies report improvement in at least one cardiovascular or vascular parameter, robust clinical data in humans are still lacking.

Other experimental approaches currently under investigation include phosphodiesterase type 5 inhibitors, maternal gene therapy targeting uteroplacental angiogenic pathways, and transamniotic mesenchymal stem cell therapy. In experimental models, these strategies have been associated with partial normalization of fetal cardiac growth and reductions in markers of myocardial stress and inflammation, but they remain investigational and require careful evaluation before any clinical application can be considered [134,135,136,137].

Particular attention has been devoted to phosphodiesterase type 5 inhibitors, especially sildenafil, which have been investigated in the STRIDER (Sildenafil TheRapy In Dismal prognosis Early-onset fetal growth Restriction) trials. Although early experimental and pilot clinical studies suggested potential benefits in improving uteroplacental perfusion, the STRIDER trials conducted in different populations did not demonstrate clear improvement in perinatal outcomes and raised safety concerns, including possible increases in neonatal pulmonary complications [138,139,140]. As a result, current evidence does not support the routine clinical use of sildenafil for the treatment of FGR, and its use remains limited to research settings.

In addition to pharmacological strategies, modifiable environmental and lifestyle factors deserve attention in future preventive approaches. Maternal smoking is a well-established risk factor for placental insufficiency and FGR and is associated with adverse fetal cardiovascular programming [141,142]. Furthermore, increasing evidence suggests that environmental exposures, including air pollution and fine particulate matter (PM2.5), may impair placental function and fetal growth through inflammatory and oxidative stress pathways [143,144]. Addressing these modifiable exposures may represent an important component of comprehensive prevention strategies for FGR and its long-term cardiometabolic consequences.

#### 8.1.2. Postnatal Management

Postnatal factors may further influence the cardiovascular trajectory of individuals born with FGR. In the early neonatal period, delayed cord clamping in growth-restricted neonates improves several hemodynamic and hematological parameters, including superior vena cava flow, right ventricular output, and iron stores, without a clear increase in short-term adverse effects [145].

Nutrition and growth patterns during infancy and childhood appear to play a central role in modulating long-term cardiovascular risk. Breastfeeding for more than six months has been associated with more favorable indices of left ventricular geometry, and healthier fat intake in early childhood with improved carotid intima–media thickness. In contrast, excessive catch-up growth and the development of overweight or obesity amplify vascular remodeling in children born with FGR [146].

Given the evidence of early and persistent cardiovascular remodeling, structured cardiovascular follow-up may be justified in selected high-risk individuals. A composite fetal cardiovascular risk score based on functional and hemodynamic parameters has shown good predictive value for early postnatal hypertension and arterial remodeling, although such tools are not yet part of routine clinical practice [147].

### 8.2. Research Gaps and Future Directions

#### 8.2.1. Critical Knowledge Gaps

Despite the growing body of evidence, several fundamental questions remain unresolved. Although structural and functional cardiovascular alterations are consistently observed in FGR fetuses, neonates, and children, their long-term trajectory remains incompletely defined [84,147]. It is still unclear to what extent these changes persist, progress, or partially normalize across the life course. While epidemiological studies demonstrate an association between low birth weight and increased cardiovascular morbidity and mortality in adulthood, the causal and mechanistic links between early-life cardiac remodeling and later clinical disease remain largely inferential [124].

Another major limitation is the absence of standardized postnatal screening and management strategies. Although subtle echocardiographic abnormalities and biomarkers of myocardial stress can be detected early in life, there is currently no consensus regarding optimal screening intervals, clinically meaningful thresholds, or evidence-based interventions for asymptomatic individuals born with FGR [15,124].

#### 8.2.2. Future Research Priorities

A major priority is the development and validation of sensitive and reproducible tools for the early detection of subclinical cardiovascular involvement. Advanced imaging techniques, including myocardial deformation analysis and vascular stiffness assessment, together with circulating biomarkers of myocardial stress, fibrosis, endothelial dysfunction, and metabolic remodeling, may enable improved risk stratification long before the clinical manifestation of disease and support the implementation of targeted preventive strategies [15,124,148].

Longitudinal studies across childhood, adolescence, and early adulthood are needed to define the natural history of cardiovascular remodeling in individuals born with FGR and to clarify whether early abnormalities persist, progress, or partially normalize over time. Such studies will be crucial to identify critical windows of vulnerability and potential opportunities for early intervention.

Ultimately, integrating experimental, clinical, and epidemiological data will be essential to achieve a more mechanistic and clinically actionable understanding of cardiovascular programming in FGR, paving the way for personalized surveillance, prevention, and early therapeutic strategies in this high-risk population.

Importantly, future research should frame cardiac and vascular findings within a broader physical health context. FGR-related cardiovascular remodeling does not occur in isolation but is part of a multisystem developmental programming process that also involves renal function, glucose metabolism, insulin resistance, type 2 diabetes risk, and the development of metabolic syndrome. An integrated life-course approach that considers cardiovascular, renal, and metabolic outcomes together may better capture the full clinical impact of FGR and improve risk stratification and long-term preventive strategies.

## 9. Strengths, Limitations, and Clinical Implications

This review provides an integrated life-course perspective on the cardiovascular consequences of fetal growth restriction, combining experimental, imaging, and epidemiological evidence from fetal life to adulthood. By synthesizing mechanistic and clinical data, the manuscript offers a coherent framework linking placental insufficiency, myocardial remodeling, vascular alterations, and long-term cardiovascular vulnerability. The integration of structural, functional, and metabolic findings allows a comprehensive interpretation of FGR as a systemic developmental condition rather than a purely obstetric diagnosis.

Several limitations should be acknowledged. As a narrative review, this work does not include a formal risk-of-bias assessment or quantitative meta-analysis, and although the literature search was structured and transparent, some degree of selection bias cannot be excluded. The available studies are heterogeneous in terms of FGR definitions, study design, imaging methodologies, and duration of follow-up, limiting direct comparability and precluding firm causal inferences. Moreover, many human studies include relatively small cohorts or mixed populations (e.g., preterm and growth-restricted infants), making it difficult to isolate the independent contribution of fetal growth restriction from other perinatal factors.

Overall, the available evidence suggests that FGR may represent an early marker of increased cardiovascular susceptibility. From a clinical perspective, this supports adopting a life-course approach to follow-up, particularly in individuals with severe or early-onset FGR or documented fetal hemodynamic abnormalities. While standardized screening protocols are not yet established, greater awareness of potential long-term cardiovascular and cardiometabolic implications may justify individualized monitoring of blood pressure, growth patterns, and metabolic risk factors during childhood and early adulthood. Further longitudinal studies will be essential to refine risk stratification and inform evidence-based preventive strategies.

## 10. Conclusions

Fetal growth restriction represents a complex developmental condition with significant implications for long-term cardiovascular health. Beyond its association with adverse perinatal outcomes, current evidence indicates that an adverse intrauterine environment may influence myocardial development and vascular structure during critical windows of cardiac maturation, potentially shaping cardiovascular vulnerability later in life.

Although the precise mechanisms and long-term trajectories remain incompletely defined, the available experimental and clinical data support interpreting FGR within a developmental programming framework. Recognition of FGR as a potential early cardiovascular risk condition underscores the importance of accurate diagnosis and careful differentiation from constitutional smallness.

Future progress will depend on longitudinal studies, improved phenotypic characterization, and integration of cardiac, vascular, and metabolic outcomes within a life-course model. Such an approach may ultimately facilitate earlier risk identification and more targeted preventive strategies, with the aim of reducing the long-term cardiovascular burden associated with fetal growth restriction.

## Figures and Tables

**Table 1:** Key human studies on cardiovascular outcomes in fetal growth restriction/small for gestational age.

Reference	Population	Study Design	Main Findings
Fouzas et al. [119]	IUGR neonates	Observational	↑ BNP levels; ↑ MPI (subclinical biventricular dysfunction)
Zaharie et al. [118]	IUGR neonates vs. AGA	Case–control	LV systolic dysfunction (MPI > 0.47 in ~40% IUGR vs. 4.7% controls); smaller LV dimensions
Bjarkø et al. [132]	FGR neonates	Observational	Reduced LV geometry at birth
Skilton [55]	Growth-restricted neonates	Comparative	↑ Aortic intima–media thickness (early vascular remodeling)
Raitakari et al. [133]	Adults born SGA	Longitudinal cohort	↑ SBP, ↑ LDL, ↑ triglycerides; ↑ carotid IMT in adulthood
Miles et al. [126]	Young adults (~21 yrs)	Cross-sectional	↑ AIx and central BP (attenuated after BSA adjustment)
Lu et al. [127]	SGA & preterm adults	Population-based cohort	HR 1.38 for CVD in SGA; prematurity independently associated
Bjarnegård et al. [129]	Young adults born IUGR	Case–control	↓ Ascending aortic diameter; ↑ AIx
Morsing et al. [130]	7-year-old children	Comparative study	Endothelial dysfunction; ↑ SBP; arterial remodeling
Tzschoppe et al. [131]	IUGR children (≤6 yrs)	Prospective follow-up	↑ Carotid IMT; early vascular remodeling

↑ indicates an increase, whereas ↓ indicates a decrease. AGA, appropriate for gestational age; AIx, augmentation index; BNP, brain natriuretic peptide; BSA, body surface area; CVD, cardiovascular disease; FGR, fetal growth restriction; HR, hazard ratio; IMT, intima–media thickness; IUGR, intrauterine growth restriction; LDL, low-density lipoprotein; LV, left ventricle; MPI, myocardial performance index; SBP, systolic blood pressure; SGA, small for gestational age.

## Data Availability

No new data were created or analyzed in this study.
